# Is a bio-psychosocial approach model possible at the first level of health services in the Democratic Republic of Congo? An organizational analysis of six health centers in South Kivu

**DOI:** 10.1186/s12913-023-10216-0

**Published:** 2023-11-11

**Authors:** Christian Eboma Ndjangulu Molima, Hermès Karemere, Samuel Makali, Ghislain Bisimwa, Jean Macq

**Affiliations:** 1 École Régionale de Santé Publique, Université Catholique de Bukavu, Avenue Michombero N°2, Kadutu, Bukavu, Democratic Republic of Congo; 2 Institute of Health and Society (IRSS), Université Catholique de Louvain, Brussels, Belgium

**Keywords:** Organizational, Person-centered care, Biopsychosocial, Health center, DRC

## Abstract

**Background:**

The health system, in the Democratic Republic of Congo, is expected to move towards a more people-centered form of healthcare provision by implementing a biopsychosocial (BPS) approach. It’s then important to examine how change is possible in providing healthcare at the first line of care. This study aims to analyze the organizational capacity of health centers to implement a BPS approach in the first line of care.

**Methods:**

A mixed descriptive and analytical study was conducted from November 2017 to February 2018. Six health centers from four Health Zones (South Kivu, Democratic Republic of Congo) were selected for this study. An organizational analysis of six health centers based on 15 organizational capacities using the Context and Capabilities for Integrating Care (CCIC) as a theoretical framework was conducted. Data were collected through observation, document review, and individual interviews with key stakeholders. The annual utilization rate of curative services was analyzed using trends for the six health centers. The organizational analysis presented three categories (Basic Structures, People and values, and Key Processes).

**Result:**

This research describes three components in the organization of health services on a biopsychosocial model (Basic Structures, People and values, and Key processes). The current functioning of health centers in South Kivu shows strengths in the Basic Structures component. The health centers have physical characteristics and resources (financial, human) capable of operating health services. Weaknesses were noted in organizational governance through sharing of patient experience, valuing patient needs in Organizational/Network Culture, and Focus on Patient Centeredness & Engagement as well as partnering with other patient care channels.

**Conclusion:**

This study highlighted the predisposition of health centers to implement a BPS approach to their organizational capacities. The study highlights how national policies could regulate the organization of health services on the front line by relying more on the culture of teamwork in the care structures and focusing on the needs of the patients. Paying particular attention to the values of the agents and specific key processes could enable the implementation of the BPS approach at the health center level.

## Background

Health systems in sub-Saharan Africa remain mostly focused on disease control and mortality reduction for targeted groups of the population (e.g. maternal and child mortality) [1]. Despite a recurrent discourse on health system strengthening for people (i.e. people-centered care) in the first line of care, most financing and performance measurement strategies are still targeting the same priorities (disease and targeted “at risk” groups) in primary healthcare facilities like health centers [2, 3].

In the Democratic Republic of Congo (DRC), the health system is organized by the national Ministry of Health (central level), the province as intermediate level, and each one is organized at the operational level in health zones (HZ). The central level of the Ministry of Health in Kinshasa has a normative role. The intermediate level is in charge of technical and logistic support to health zones by the provincial health division managing. The operational level comprises 516 health zones (HZs) subdivided into health areas (HAs). HZ is managed by a health zone management team led by the chairman of the Health Zone medical chief in collaboration with the health zone management committee extended to non-healthcare providers. Health centers (HC) are expected to provide primary care in each HA through a package combining curative services, prevention, and health promotion. Community participation is effective by health committees extended to non-healthcare providers at the health center level. Complementary more technical care is provided at Reference health centers and Referral Hospitals offering inpatient and reference services (e.g. dystocic deliveries, surgeries, blood transfusions, medical imaging, and specialized medical imaging examinations). The medical team of Referral Hospitals assumed technical support to health centers through supervision as members of the health zone management team [4–6].

Public primary care services governance still follows a command and control approach as required by international donors [7]. This conditions data collection, indicators definition, and their use for accountability rather than learning and eventual adaptation of services.

International voices have called, for mainstream approaches to move towards a more holistic, people and community-centered form of healthcare provision. The purpose of healthcare should be to contribute to individual and community health as part of an overall development process [8, 9].

In other words, this would imply considering more than the physical or biological aspects by integrating the functional, cognitive, and social aspects in the support of people within the community. The biopsychosocial model of care is an approach to healthcare that recognizes the importance of addressing biological, psychological, and social factors in the assessment, diagnosis, and treatment of disease and illness. This model acknowledges that these three factors are interconnected and that addressing them together can improve health outcomes for patients [10].

The patient-centered or person-centered approach is a criterion of quality of healthcare that prioritizes the patient or person's needs, values, preferences, and perspectives in all aspects of care, including diagnosis, treatment, and management [11, 12].

The biopsychosocial model as an application of the person-centered approach represents a shift away from the traditional biomedical model of healthcare, which focused solely on biological factors, and toward a more holistic and person-centered approach that recognizes the importance of addressing persons' unique needs and perspectives in healthcare [13, 14].

To develop this so-called biopsychosocial approach (BPS), which is part of the person-centered approach, the health center team seems best positioned to adopt this way of providing care. Nevertheless, this requires changes both at the organizational level and in the professionals working in this structure.

Indeed, the person-centered care approach, from which bio-psycho-social care emerged, is one of the attributes of quality care [11, 15]. Patient-centered care is derived from the quality of personal, professional, and organizational relationships. Thus, efforts to promote patient-centered care should consider attention to patients (and their families), clinicians, and health systems [16, 17], including the health center in our study. Core characteristics of the person-centered care approach have been identified as patient involvement in care and individualization of patient care [18].

The term 'patient-centered' approach to medical care thus refers to a style of practice that is oriented towards the needs of the patient rather than the agenda of the health care provider and thus moves from professional control to patient empowerment. Its main components are patient-centered interviewing and patient counseling [19, 20]. The BPS approach to care takes the process of patient-centered counseling a step further, going in our study beyond the concept of "patient" to the concept of "person" which is more inclusive of other aspects than medical. The BPS approach builds on observations that psychosocial factors are determinants of health [21] and on Engel's BPS model [22–24] which assumes that patient complaints cannot be considered in isolation from their psychosocial causes and consequences. Therefore, a BPS orientation is an effort to gain insight into both the biomedical and psychosocial aspects of the person’s predicament to help them manage them simultaneously [25]. The person-centered by BPS approach to care has been shown to improve patient satisfaction, reduce the frequency of malpractice suits, and improve health outcomes [19].

Efforts to implement a BPS approach to care may encounter barriers or facilitating factors [26]. Disadvantageous factors identified included providers’ differing understanding of the approach as a mental health program and facilitating factors included home visits and the dynamism and leadership of the care team [26].

 To change the form of healthcare provision at the health center level, it’s important to examine whether and how changes are possible in the approach to prioritizing, organizing, and providing healthcare at the primary healthcare level (health center) toward more people-centered care and community health. A large development research project funded by the Académie de Recherche et d'Enseignement Supérieur (ARES) in South Kivu (eastern DRC) aims to assess the feasibility of implementing a package of interventions in health centers to shift to the BPS approach to care. On the initiative of Belgian academic institutions (Université Catholique de Louvain and Université Libre de Bruxelles), the promotion of a BPS management model in the first line of care was proposed as a fundamental element of the research to be carried out in collaboration with the Université Catholique de Bukavu and the Ministry of Health (MoH).

The objective of this study is to analyze the organizational capacity of the health centers to implement a BPS approach and to propose a package of interventions for a BPS approach to be implemented in the analyzed health centers.

## Methods

### Study settings

South Kivu, in the Eastern part of DRC, has 34 HZs, which are considered the operational level of the health system. Each HZ is divided into HA (15 on average) and organized around an HC (primary healthcare level of the DRC health system). Six HCs (2 urban and 4 rural) from four HZs were selected for this study: Nyamuhinga and Lumu HCs in Bagira, Bideka and Burhale HCs in Walungu, Lwiro HC in Miti-Murhesa and Kabushwa HC in Katana. This choice was based on their accessibility and their partnership with non-governmental organizations (Louvain Cooperation) that is also part of a broader project on which this study draws. The geographical location of the health zones was taken into account for their selection (to the north-east the health zones of Katana and Miti-Murhesa, to the south-west the health zone of Walungu, the health zone of Bagira in the town of Bukavu on the banks of Lake Kivu) by the research team, technical staff from the Provincial Ministry of Health and from NGO Louvain Cooperation. At the time of the study, the Nyamuhinga Health Center was receiving institutional support from the NGO Louvain Cooperation, which consisted of the rehabilitation of the infrastructure, the supply of essential medicines, and the payment of operating and motivational bonuses for the health center staff. Technical support and an operating allowance were provided to the health zone management team.

**Fig. 1:**
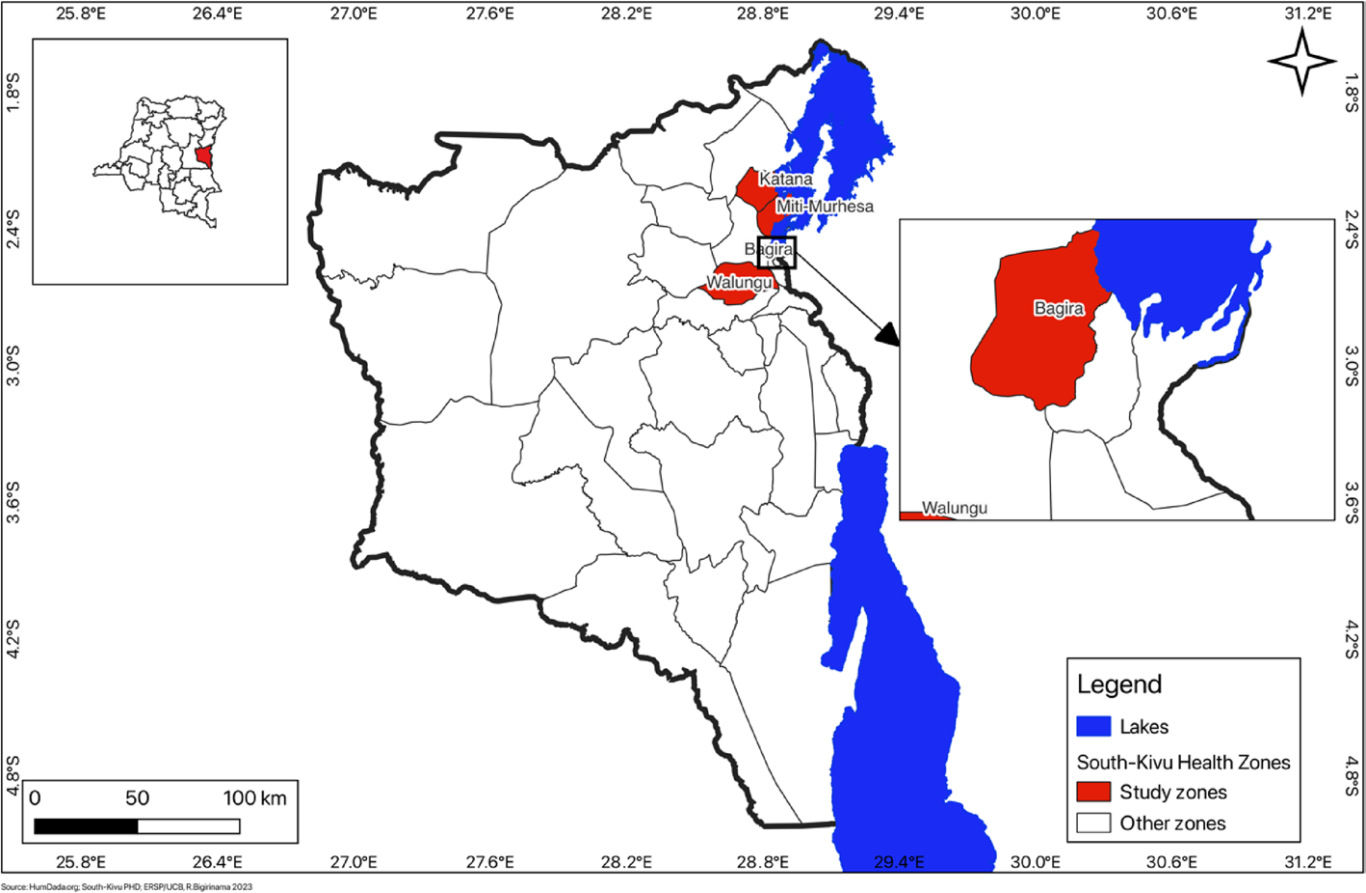
Health zones concerned by organizational analysis in South Kivu, DRC (2017–2018)

Table 1 below describes the characteristics of the six health centers selected in our study. Figure 1 below describes the study environment in which our research was conducted.

**Table 1:** Characteristics of the six health centers involved in the organisational analysis

Health Center	Health Zone	Number of employees	Qualification	Type of care facility	Localization
Bideka	Walungu	12	Nurses Midwives Laboratory assistant	Confessional structure	Rural
Burhale	Walungu	12	Nurses Midwives Laboratory assistant Nutritionist	Confessional structure	Rural
Kabushwa	Katana	13	Nurses Midwives Laboratory assistant Nutritionist	Public structure	Rural
Lumu	Bagira	13	Nurses Midwives	Public structure	Semi-rural
Lwiro	Miti-Murhesa	11	Nurses Laboratory assistant	Public structure	Rural
Nyamuhinga	Bagira	13	Nurses	Public structure	Semi-rural

### Study design and period

We conducted a mixed descriptive and analytical study from November 2017 to February 2018. The study performed an organizational analysis of six health centers based on the organizational capacities of the health facilities to perform tasks that support person-centered care from a BPS perspective [27].

In 1977, Engel published a seminal article presenting a BPS model based on systems theory and the hierarchical organization of organisms [22]. In medicine, this model or BPS approach aims to take into account psychological, social, and biological factors of diseases and often requires a multidisciplinary approach.

Starting from the BPS approach, it should be determined which model of analysis of the organization of care allows for the best analysis of health centers in South Kivu, DR Congo, for the best implementation of this approach. Implementation researchers have a wide range of theoretical tools useful for analyzing the organization of care [28–30]. In this study, we used the Context and Capabilities for Integrating Care (CCIC) as our theoretical framework. This framework covers the organizational analysis of integrated care [27] and takes into account the focus on patients (and their families), clinicians, and health systems that characterize the person-centered care approach [16, 31], or BPS.

In this 17-component CCIC analysis framework [27], adapted to the present study, the organizational analysis focuses on 15 components grouped into three parts. The two components not included in the adapted theoretical framework (Table 1) are *the Leadership Approach* and *Readiness for Change*. These components are analyzed in another complementary study dedicated to the analysis of mindset transformation in the six health centers analyzed among health workers. Table 2 presents the different concepts, their definition, the subcomponents explored during the study, and the link of each subcomponent with the BPS approach at the health center level.

**Table 2:** Organizational analysis framework for health centers integrating the BPS approach (Adapted from Jenna's CCIC Framework) ( 27)

Component	Definition	Subcomponents applied to HC	Link to BPS approach for HC
***Basic Structures***			
**Physical Features**	Structural and geographic characteristics of the organization/practice and network	Appropriate consultation room, availability of staff	Ability to respect the confidentiality and privacy of patients guaranteed in the consultation room
**Resources**	Availability of tangible and intangible assets for ongoing operations at the organization/practice and for network activities	Availability of means to organize the supply of care	Capacity to mobilize resources to implement the BPS approach
**Governance**	How the board or steering committee is organized and its activities to direct, manage and monitor the affairs of the organization/practice and network	Experience sharing, communication on service management	Ability to implement a better organization of services integrating the bio-psychosocial aspects of care
**Accountability**	The mechanisms in place to ensure that people and organizations meet formal expectations in the organization/practice and network	Supervision of health centres by the Health Zone Management Team and partners, functioning of the health committee	Capacity for accountability and supervised responsibility of the health center team in the implementation of approaches, including BPS
**Information Technology**	The availability and ease of use of technology-based communication and information storage mechanisms in the organization/practice and across the network	Community members’ Patient record, Computerization of personal data	Ability to collect information on the biomedical, psychological and social characteristics of community members
**Organizational / Network Design**	The arrangement of units and roles and how they interact to accomplish tasks in the organization/practice and network	Communication for coordination and continuity of care, existence of community support groups	Ability to organize continuity of care and psycho-social support for patients at community level
***People and values***			
**Clinician Engagement & Leadership**	The formal and informal roles held by clinicians in the organization/practice and network, particularly physicians, that enable them to buy-in to and steer change, and influence others	Mentoring of health center teams by doctors from the Health Zone on the BPS approach	Ability to influence change, by promoting the practices of caregivers and the exchange of information between the HC and the general referral hospital for a better management of BPS issues
**Organizational / Network Culture**	Widely shared values and habits in the organization/ practice or network	Shared definition of the BPS approach within the team; shared vision and values about patient-centered care and the BPS approach	Ability to structure the care offer according to the BPS approach, influenced by the values and habits of the care teams
**Focus on Patient-Centeredness & Engagement**	Commitment to placing patients at the center of clinical, organizational and network decision-making	Taking into account patients' impressions and preferences in the organization of care	Ability to integrate patient preferences into the provision of care, using the BPS approach
**Commitment to Learning**	The existence of a set of values and practices that support ongoing development of new knowledge and insights within the organization/practice and network	Promotion of new practices, participation in training sessions and case discussions	Ability to implement new practices including those related to the BPS approach
**Work Environment**	How employees perceive and experience their job and their workplace in the organization/practice and network	Collaboration between team members, Job satisfaction	Ability to organize collaborative, interdisciplinary and multidisciplinary work compatible with the BPS approach
***Key processes***			
**Partnering**	The development and management of formal and informal connections between different organizations/practices	Sharing of experiences and personnel, Referrals of people and sharing information	Ability to interact with formal and informal partners to strengthen the implementation of the BPS approach
**Delivering Care**	The methods used by providers in caring for patients in the organization/practice and network	Use of flowcharts in care and the BPS model, nature of patient-caregiver relationships	Ability to structure the provision of care according to the BPS approach and to support it with tools based on scientific evidence, ability to build better patient-caregiver relationships
**Measuring Performance**	The systematic collection of data about how well the organization/practice and network is meeting its goals	Measurement of indicators, Preparation of regular reports (timeliness and completeness), Access to data	Ability to measure the performance of the health care offer according to a BPS approach, to document the events and practices related to this approach in order to improve and achieve objectives
**Improving Quality**	The use of practices and processes that continuously enhance patient care in the organization/practice and network	Improving the quality of care, Application of best practices	Ability to manage change to improve the quality of care delivery using the BPS approach

### Data collection

The study used quantitative data, mainly the number of new cases per health center and total population of health areas over the period 2013 to 2016, as well as qualitative data.

These data were collected using three techniques: observation during visits to health centers, document review, and individual interviews with zone chief medical officers, health care providers, and members of health and development committees.

A non-participatory direct observation was conducted by the principal investigator in each of the health centers. Based on a framework designed from the CCIC components, data on the physical characteristics of the health centers, the organization of the patient file within the health facilities, and the health information system used by the health center staff were reported.

The principal investigator conducted the literature review over three days per health center. The annual reports of the health centers from 2013 to 2016 and the minutes of the CODESA (health and development committee of the health area) meetings kept in the health structures were used. The data was collected on the basis of a framework comprising statements on the resources of the health centers (human, financial, material, and inputs including medicines), the governance structures in the health center, the clinical information system, the managerial health information system, the relations of the health center with the other health care providers in the health area, continuous training, and the quality of the reinforcement of the health center. The level of the care activity package and the care package offered at the health center was reported through the National Health Information System (SNIS) indicators from 2013 to 2016, including new cases per month during this period.

The individual interviews involved the head nurses of the health centers, their assistants, representatives of CODESA, and the Health Zone medical chief of the 4 health zones that comprise the 6 health centers involved in the study. In total, 16 people participated in the interviews as key informants.

The principal researcher conducted the interviews in French in a room available within the health centers or the health zone office and recorded using a Dictaphone. The participants gave their written consent after the principal investigator had explained the aims of the research in detail, specifying that they had voluntarily chosen whether or not to take part. An interview guide inspired by the analysis framework adapted from J. Evans' CCIC framework (Table 2) was used, covering the components of the three categories, namely structural elements, human elements, and key processes of the health centers.

### Data analysis

Concerning the quantitative aspect, trends in the annual utilization rate of curative services in health centers were analyzed using data from the literature review in the six health centers. This indicator was chosen to highlight the fact that the health centers were offering the primary care provided in the first line of care package at the time of our study. We consider this data to be an indicator of the ordinary functioning of the health center, a prelude to reflections on the change in the content of the offer in the first line of care. The different rates were compared based on the benchmarking method using a scale based on “poor” for a rate below 30%, “fair” for a rate between 30 and 49%, “good” for a rate between 50 and 79% and “excellent” for a rate of at least 80%. The data is reported in a bar chart by center and by year and presented based on an average of 50% in good performance (above 50%) and poor performance of curative services (below 50%) [32].

Concerning the qualitative aspect, the organizational analysis consisted of a specific evaluation of the organization of the HCs about the BPS approach using data from observations and in-depth individual interviews conducted with key informants. Based on the sub-components retained in the analysis framework and the interview guide (Table 1), the information was grouped into three main themes with sub-themes: (1) structural elements that could influence a BPS approach to caring for people, (2) human elements and informal processes that could influence a BPS approach to caring for people, and (3) key formal processes that the HC would need to engage in to influence a BPS approach to care for people [27].

For each theme, strengths and weaknesses are presented by the health center and supported by data from key informant interviews (IC1, IC2, … IC8). By deductive thematic analyses, the analysis is presented first globally in the form of a graph comparing the health centers with each other and then by the theme of organizational analysis, taking into account the information from the interviews and observations. For each category of components, the gaps are identified at the organizational level for the implementation of the BPS approach in each health center.

## Results

The results of our study are presented in two groups. The first group, on the annual utilization rate of curative services per health center, presents the results of the quantitative approach. The second group, relating to the health centers' organizational analysis, describes the qualitative approach's results.

### Changes in health center utilization between 2013 and 2016

The trend in annual rates of use of services by health centers shows that in 2013 the six health centers performed poorly in terms of curative services, with average rates in Lwiro and Kabushwa.

**Fig. 2:**
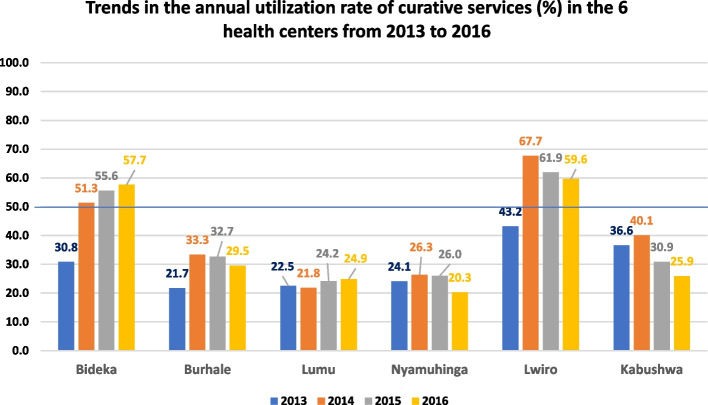
Trends in the annual utilization rate of curative services (%) in the 6 health centers from 2013 to 2016

From 2014 to 2016, only the Bideka and Lwiro health centers performed well in using curative services in their facilities. Figure 2 below describes trends in annual rates over time by center.

### The overall organizational capacity of the health centers to implement the BPS approach

A. Basic structures

Regarding the physical characteristics of the health centers, they all have functional consultation rooms, which is a strength. However, the health centers of Lwiro and Nyamuhinga do not have the comfort and privacy necessary for a BPS approach in the consultation room. The availability of human resources at the health center and of a functional building are noted by key informants as basic elements for the implementation of a BPS approach.

*"As far as the basic criteria are concerned, I would say that first of all, we need a sufficient number of qualified and well-trained staff for biopsychosocial care; secondly, we need to have adequate infrastructures that allow us to provide this correct care; we need to have activities in the community to provide this BPS support"* (IC3).

Concerning resources, the health centers mobilize the funds necessary for their operation thanks to fundraising and the support of certain partners. Only the health centers in Lumu and Nyamuhinga receive additional subsidies from the government, which is a strength. For some key informants, the support of partners for health centers can guarantee the implementation of a BPS approach according to certain prerequisites.

*"For a partner to be able to support the implementation of BPS care, it is, first of all, a question of infrastructure, having a room where the consultation can take place calmly,… and also equipping the health center with materials and medicines, and also motivating the health team" (IC8).*

Regarding Governance, the teams of the health centers of Burhale, Kabushwa, Lumu, and Lwiro do not share their experience of patient monitoring in staff meetings, which is a weakness.

The supervisions organized by the central offices of the health zones are evaluative for new practices in Bideka, Burhale, and Nyamuhinga although the supervisors of three health zones (Bagira, Walungu, and Miti-Murhesa) do not know the BPS approach, which is a weakness in accountability. This aspect is important to consider according to some key informants:

*"If we have already internalized the approach at our level, this will *constitute* supervision modules, when we carry out field supervision visits, we will also evaluate their programming of activities, we will see where they have inserted support by making home visits or even listening sessions for psychological care" (IC1).*

*"How the health zone can get involved in the medical-psycho-social problem in the health center is to accompany the *providers* at the health center level, in continuous training and this through supervision. It can look for funding partners so that the activity is effective" (IC8).*

The patient file used in the health centers does not exist in electronic format and only in Nyamuhinga is it possible to access the BPS data of people using health services, which is a weakness. These records are securely filed and the data is accessible in the different health centers in the shape of Health cards, notebooks, and Patient files, a strength reported in Information Technology.

In Organizational / Network Design, the health center data reports that there are solidarity groups (mutualities, patient groups, savings, and credit associations) in the health areas of Bideka, Kabushwa, Lwiro, and Nyamuhinga, and these collaborate with the health center staff, which is a strength, especially as the center staff is involved in setting up some of them.

*"They certainly have a big role to play; first of all, they allow access to care *and* this is more in line with the solidarity aspect, so in short, it's really social, and in my opinion, these structures can really help if the partners withdraw to facilitate access to care" (IC3).*

*"We are also part of the community, when these solidarity mutualities are *there*, we are also beneficiaries and facilitators, there are others that we have even initiated at the level of the structure here, the mutuality that we call AVEC (Association Villageoise d'Épargne et de Crédit), …" (IC5).*

B. People and values

Regarding Clinician Engagement and Leadership, the reference level doctors in the four health zones report knowing the BPS approach but without mentoring the health center teams, a weakness noted in the six health centers.

*"Our competence is limited because of the information we have, I am a doctor … and I used to focus on the clinic only and I used to suggest to the patient what he can take as medicine without soliciting his opinion because I am aware that our way of managing the patient in our facilities is not yet up to the mark because there are shortcomings although we may have theoretical training we don't manage to practice"(IC2).*

In the majority of health centers, staff members do not have a formalized definition of a BPS approach at the health center and do not share a common value in terms of a person-centered and rights-based vision. This weakness noted in Burhale, Kabushwa, Lumu and Lwiro in Organizational / Network Culture indicates that the team's values and habits do not yet integrate the BPS approach in the provision of care. Nevertheless, the need to change the way of working is underlined by some key informants: *"Yes—it's necessary at a certain level because the HC agents are not used to doing the care as it is defined by the psychological, medical and also social part; so it's a new approach. (IC1).*

The Organizational/Network Culture in some health centers takes into account the context of the community members by ensuring an adapted care offer: *"Our service never ends… we have taken another way of working which may not suit other structures… everyone works during the day and it is only after 4 pm that one person is on duty until the next day when everyone works… with us, all activities are functional every day except Sunday" (IC5).*

In all the health centers except Nyamuhinga, there is no system for collecting patients' impressions of the organization of care. Nevertheless, in Bideka, Burhale, Kabushwa and Nyamuhinga, the health center teams communicate with people during the consultation about their values and preferences in terms of care. This positively influences the Focus on Patient- Centeredness & Engagement in these facilities.

The members of the health center teams did not receive training on the BPS approach in their academic curriculum and no evaluation was organized for those who received training in this approach in the six health centers, which negatively influences Commitment to Learn. According to some key informants, the training of health center staff would be an asset:

*"The implementation of this BPS approach can add some empowerment, we will detect their limit and at our level, we will do advocacy for empowerment sessions* *even training and that will be a plus." (IC1).*

*"… I told you that the empowerment of the agents would be welcome because to say that all the cases that we can believe to have BPS problems can be for example* *referred to another person and that can't make it easier for us to take care of them…" (IC7).*

For others, it should not concern all agents involved in care. *"It would be better if it concerned all the agents but as we all know, the means are limited because with all the agents it will require a lot of means whereas they are limited and therefore it would be better to take the persons in charge and that they can go and do a restitution in their structure" (IC2).*

*"We have received training, it's true, but it should be continuous because there *are* new things every day, especially in health, … Continuous training for staff would really be an asset and also inform other people" (IC5).*

Only the Nyamuhinga team organizes case discussions oriented towards the BPS management of people by proposing a plan for implementing the recommendations, which is a strength reported in this facility. In the other health centers, staff meetings mainly concern the medical follow-up of people under observation in the health facilities.

*« Our meetings are much more about medical issues because this bio-*psycho*-social approach is being integrated but has not been fully integrated yet… but when it will be very integrated, it will follow the same rhythm» (IC5).*

Concerning the Work Environment, the members of the various health centers integrate diversity (multidisciplinary) into the profile of the health center care teams, a strength noted in the six health centers since it ensures collaboration and interdisciplinarity in the health center staff.

C. Key processes

In four of the health centers, partnering is still a weakness as health center staff do not organize exchanges of experience with other facilities in the health area. They also do not share information on complex BPS cases followed up in their health facilities as noted in Burhale, Kabushwa, Lumu, and Lwiro.

Regarding Delivering Care, the staff of Kabushwa, Lwiro and Lumu health centers do not communicate with each other and do not share their experiences, which is a weakness. According to some key informants, the management provided by the strong leadership of the Head Nurses or IT could influence the teams in the health centers.

*"I think that even the leadership of the nurse in charge of the health center must be very much called upon if we are to succeed and if the whole team is to understand the need for this care, which is holistic and which takes the human being as an individual and not as a medical aspect. (IC3).*

With regard to Measuring Performance, all the health centers report quantitative indicators on the state of the community's health, and reports on the functioning data of the health centers and their indicators are available in the health facilities. Only Nyamuhinga reports the psychosocial results of consultations carried out in its center.

For Improving Quality, only the Bideka and Nyamuhinga teams are making changes in the organization and provision of care within their facilities. This change could be more strongly felt under certain conditions: *"If the ITs or those in charge of these HCs are very involved, they will take on the task of accompanying the other members of their team to implement the approach because if they have already understood at the outset, things will go smoothly in my opinion" (IC1).*

This change can be assessed either through a reporting and feedback system implemented by the health center teams (as in Bideka, Burhale, Kabushwa, and Nyamuhinga) or by the health zone management team. *"Evaluation can happen at two levels in my understanding; the first level is through supervision and supervision is defined by the review and the second level is surveys and performance evaluation at the facility level. (IC2).*

## Discussion

The objective of this article was to analyze the organizational capacity of the health centers to implement a BPS approach and to propose the intervention package of a BPS approach to be implemented in the analyzed health centers.

Based on our results, it appears that there are strengths and weaknesses in the health centers' capacity to implement the BPS approach. Some strengths include the availability of functional consultation rooms, mobilization of funds for operation, the presence of solidarity groups, and the integration of diversity in the health center care teams. However, there are also weaknesses such as the lack of comfort and privacy necessary for a BPS approach in consultation rooms in some health centers, the lack of sharing patient monitoring experiences in staff meetings, supervisors' lack of knowledge on the BPS approach, no system for collecting patients' impressions of the organization of care, lack of formalized definition of the BPS approach, and the absence of a training program and evaluation for the health center staff on the BPS approach.

Using Jenna's adapted framework (Table 1) and benchmarking methods, we observed that the different care structures are functioning effectively in providing care services to members of their community while reflecting identified gaps per health center and by component for *the Basic structures, people and values, and Key processes*. The ideal organizational level for the implementation of the BPS approach is not reached in the health centers analyzed.

For the Basic structures, the results of the analysis show that the majority of health centers report an insufficiency in the application of Governance regarding the sharing between staff members on the follow-up of people. In People and Values, several health centers reported marked deficits in Organizational / Network Culture and in Focus on Patient-Centeredness and engagement. Regarding the Key Processes, several health centers have not sufficiently developed Partnering with other healthcare structures as well as Delivering Care and Improving Quality in the health centers.

Analysis of data from the interviews, literature review, and observation of the health centers shows that the organizational capacities associated with the domain of "People and values" are the least developed by the HCs to foster the implementation of the BPS approach. Studies suggest that these capabilities namely Clinician Engagement & Leadership [33], Organizational / Network Culture [34], Focus on Patient-Centeredness & Engagement [34, 35], Commitment to Learning [35, 36] and Work Environment [37] should not be overlooked in the development of the person-centered care approach. Not considering the BPS approach in the normative organization of care works against the engagement of clinicians, the change of organizational culture, and the commitment to learning the BPS approach in the different health centers. In addition, the lack of formalized definition and common values regarding the biopsychosocial (BPS) approach among health center staff, as noted in our study, is a common challenge faced in many developing countries [38, 39]. This can lead to a lack of consistent and integrated care that takes into account the person as a whole, including their psychological and social needs [40]. Therefore, it is important to promote the adoption of the BPS approach as a core value in healthcare and to provide training and support to health center staff to ensure consistent implementation.

Qualitative studies on person-centered care also highlight the importance of Physical Features [41], Resources [42], Governance [43–45], Accountability [34, 46], Information Technology [47] et Organizational / Network [34] which are organizational capabilities under the domain "Basic structures" domain. Alongside the other components, the physical characteristics of the health centers stand out as an important structural element to be considered for the implementation of a person-centered approach since they facilitate the continuity of care. The availability of functional consultation rooms is a basic requirement for effective healthcare delivery, and the lack of comfort and privacy can hinder the implementation of a biopsychosocial approach, as noted in our study. This finding is consistent with the literature, which emphasizes the importance of physical infrastructure in healthcare provision. A study conducted in rural Uganda found that the availability of functional consultation rooms and human resources was important for the implementation of a patient-centered approach in primary care. Additionally, the study found that a lack of privacy and comfort in consultation rooms can be a barrier to delivering quality care [48]. The first line of care, as a point of contact with the population, is called upon to meet certain criteria in terms of the buildings and premises to be used for care activities. As the Ministry of Health's standards stipulate, medical care is possible in various health centers. The presence of premises adapted to preserve people's privacy can facilitate follow-up activities and social care in functional healthcare structures.

The financing of health structures is still a limiting factor in the implementation of care that involves a network for sharing and supporting experiences and supporting community members. The health centers are supported by funds from external donors, but this funding is most often directed towards the organization and support of specific care activities. In addition, health center workers are not regularly paid by the government, which would make them more interested in other financial incentives [49]. In terms of resources, the fact that health centers mobilize funds through fundraising and partner support is not uncommon, particularly in resource-limited settings where governments may not be able to fully fund health services. The literature suggests that community participation and partnerships can enhance the sustainability and effectiveness of health centers [50, 51]. However, the availability of funding can be inconsistent and unreliable, leading to challenges in maintaining consistent quality of care.

Regarding governance, the lack of experience sharing in staff meetings is a weakness that has been observed in other studies as well [52]. Effective communication and collaboration among healthcare providers are essential for improving the quality of care. Studies have found that effective supervision and monitoring of health centers by higher-level authorities can improve accountability and quality of care [53, 54]. However, challenges in communication and coordination between health center staff and higher-level authorities can hinder the effectiveness of such efforts. In addition, developing a new approach to front-line care, such as in our case a BPS model based on a person-centered approach, will be easier to achieve with the support and coordination of the various hierarchical levels, such as the health zone office and the provincial division of the health zone. This support from the hierarchical level can be provided in practical terms through the supervision of health structures, a practice already underway at health centers in DRC. Several studies have shown that the implementation of innovative initiatives in the provision of care has had a real impact when the health authorities take ownership of them and support the level of provision [55–57].

Organizational / Network design in the form of health mutuals or solidarity groups supports the implementation of a BPS approach by promoting the use of health services and financial accessibility [58]. The collaboration between health center staff and community groups is also a strength that has been observed in other studies [59]. Community engagement and partnerships can enhance healthcare provision and promote health promotion and prevention initiatives.

The media used in some health centers to store information on patients and their illness episodes can facilitate patient follow-up and the organization of psychosocial support when needed.

With the help of these tools, health center teams should ensure that the confidentiality of individuals is respected when organizing multidisciplinary follow-ups of patients with psychosocial problems [60, 61]. The integration of diversity into the profile of health center care teams is indeed an important strength that promotes collaboration and interdisciplinarity. This is consistent with the general literature, which emphasizes the importance of interdisciplinary and collaborative teamwork in healthcare delivery, particularly in low-resource settings where there are often limited staff and resources [62]. Research has shown that a multidisciplinary approach to healthcare can improve patient outcomes, increase efficiency, and enhance the quality of care provided [63].

In the area of Information Technology, the absence of electronic patient records is a challenge that is also commonly observed in resource-limited settings [64]. The use of electronic health records can improve the quality of care by enabling better tracking of patient information and continuity of care.

Finally, studies show the importance of the components of the "Key processes" domain in the implementation of the BPS approach and in particular Partnering [65, 66], Delivering Care [67], Measuring Performance [68, 69] and Improving Quality [18, 68]. In our study, it was noted that there is a lack of communication and sharing of experiences among health center staff in some of the health centers, which is a weakness. This is consistent with the literature that highlights the importance of effective communication and collaboration among healthcare teams to improve patient outcomes [70].

Collaboration with other healthcare providers and alternatives to modern medicine could ensure the success of a person-centered approach by enabling good coordination of care at all levels of the health system while ensuring good quality of care for community members [71].

Health center teams can certainly act to improve quality by insisting on interdisciplinarity between staff members and improving the leadership of the various health center managers supported by the other agents. Strong leadership motivates the implementation of an approach and thus consolidates its implementation [72].

Patient-reported information is probably the best way to measure the person-centered approach and its outcomes. Including patients as key informants would have been in the best position to determine whether the care they receive corresponds to their values, preferences, and needs [31].

Moreover, only the patient knows whether he or she has received the desired level of information and whether the information is understood and can be recalled. Concerning physical comfort, only patients can also report the severity of physical symptoms and their adequate relief from medication. The use of patient-reported measures of patient-centered care is essential to identify areas of health care where improvements are needed to enhance quality [73]. In the literature, there is a strong emphasis on the importance of continuous quality improvement to enhance the quality of care, and this involves ongoing monitoring and evaluation of the care delivered [74]. Therefore, health centers need to establish a reporting and feedback system to monitor the effectiveness of changes made and to identify areas for improvement.

The components analyzed corroborate those of other studies including those of Luxford [34] and Shaller [68]. These studies reveal that several organizational attributes and processes are key facilitators to making care more person-centered including strong and committed leadership, clear communication of the strategic vision to every member of the organization, active engagement of patients and families throughout the organization, a sustained focus on staff satisfaction in a supportive work environment for all employees, active measurement and systematic reporting of patient experiences, adequate resources for care delivery redesign, staff capacity building, accountability, and incentives, a strong culture of change and learning, and the availability of supportive information technology.

Luxford mentions that the change in organizational culture from a 'provider orientation' to a 'patient orientation' as well as the time it took to move to such an orientation were the main barriers to transforming the delivery of patient-centered care [34]. Bokhour agrees, stressing the efforts that must be made at all levels of the health system on the basis that leadership must be the "primum movens" [72]. To these barriers are added those reported in our previous study [26] namely lack of knowledge of BPS management by caregivers, home visits mainly used for disease control, solidarity initiatives not promoted locally, expected new resources and financial incentives, and accountability summarized in the reporting of specific indicators.

The organizational analysis in the health centers enabled us to highlight the deficits in the current care system, particularly in terms of values and key processes, which should be considered when implementing a BPS approach in the first line of care.

However, certain limitations in relation to our findings are worth mentioning. Our study explored the functioning of six health centers to understand how care is organized according to its current model and to identify characteristics specific to each health facility that may limit or facilitate a change in healthcare provision habits.

Based on the methodology used, several limitations could impact the strengths and weaknesses of our study.

Firstly, the non-participatory direct observation method used may not have captured all aspects of the healthcare delivery process in the health centers, as it relies on the observer's interpretation of events.

Secondly, the document review of the annual reports may not have captured all the relevant information, as the reports may not have been complete or accurate.

Thirdly, the in-depth individual interviews may have introduced bias, as the interviewees may have provided socially desirable answers or may not have been fully honest in their responses.

Fourthly, the thematic analysis inspired by the CCIC framework may have missed important aspects of the healthcare delivery process that were not included in the framework.

Overall, while this study provides valuable insights into the strengths and weaknesses of the healthcare delivery process in the health centers in DRC, the limitations of the methodology used should be taken into account. Further studies using different methods or frameworks may be necessary to provide a more comprehensive understanding of the healthcare delivery process in these health centers.

## Conclusion

Based on the analysis of South Kivu health centers' organizational capacity to implement a biopsychosocial approach, several strengths and weaknesses were identified across different domains, including People and Values, Clinician Engagement and Leadership, Organizational/Network Culture, Focus on Patient-Centeredness and Engagement, Commitment to Learn, Work Environment, Key Processes, Delivering Care, Measuring Performance, and Improving Quality.

Overall, the study highlights that while there are some pockets of good practices, there are also significant gaps and challenges in the implementation of a biopsychosocial approach in health centers in South Kivu, DRC. These gaps and challenges mainly stem from a lack of standardized and formalized processes and protocols, insufficient training and mentoring of health center staff, weak communication and collaboration among health center teams, and limited patient-centeredness and patient engagement practices.

To address these challenges, several recommendations can be made to different stakeholders in the DRC healthcare organization. These include the needs to:

-Develop and implement standardized protocols and processes for the biopsychosocial approach in health centers, including training and mentoring programs for health center staff.
-Foster stronger communication and collaboration among health center teams, including sharing of experiences and information on complex cases.
-Place greater emphasis on patient-centeredness and patient engagement practices, including collecting patient feedback and involving patients in decision-making processes.
-Strengthen the leadership and management of health centers, including the Head Nurses and staff, to promote a culture of continuous learning and quality improvement.
-Ensure that health center teams are adequately resourced and supported to implement the biopsychosocial approach effectively.

In conclusion, the study suggests that improving the organizational capacity of health centers to implement a biopsychosocial approach requires concerted efforts from multiple stakeholders in the DRC healthcare organization, including policymakers, health center managers, health professionals, and patients. By addressing the identified challenges and implementing the recommended actions, health centers in South Kivu and beyond can provide more effective and person-centered care to their communities.

## Data Availability

The datasets used or analyzed during the current study are available from the corresponding author on reasonable request.

## Abbreviations

AVEC: Association Villageoise d'Épargne et de Crédit
BPS: Biopsychosocial
CCIC: Context and Capabilities for Integrating Care
CODESA: Health and Development Committee of the Health Area
DRC: Democratic Republic of Congo
HA: Health areas
HC: Health centers
HZ: Health zones
SNIS: National Health Information System
UCB: Catholic University of Bukavu
UCL: Catholic University of Louvain
